# Supplementary material for: Electrical manipulation of telecom color centers in silicon
Source: Nat Commun. 2024 Jun 3;15:4722. doi: 10.1038/s41467-024-48968-w (PMC11148098; doi:10.1038/s41467-024-48968-w)
Supplement: Supplementary file 1 — Supplementary Information [file 41467_2024_48968_MOESM1_ESM.pdf]

Supplementary Information for "Electrical Manipulation of Telecom Color Centers in Silicon"

Aaron M. Day,[1,*] Madison Sutula,[2,*] Jonathan R. Dietz,[1] Alexander Raun,[1] Denis D. Sukachev,[3] Mihir K. Bhaskar,[3] and Evelyn L. Hu[1,†]

[1] *John A. Paulson School of Engineering and Applied Sciences, Harvard University, Cambridge, Massachusetts 02138, USA*

[2] *Department of Physics, Harvard University, Cambridge, Massachusetts 02138, USA*

[3] *AWS Center for Quantum Networking, Boston, Massachusetts 02135, USA*

(Dated: May 7, 2024)

CONTENTS

I. Supplementary Note 1: Estimation of Emitter-Field Interaction 2

II. Supplementary Note 2: Fitting Information 3

III. Supplementary Note 3: Reverse Bias Extended Data 4
- A. Depletion Region 4
- B. Outside Depletion Region 5

IV. Supplementary Note 4: Forward Bias 6

V. Supplementary Note 5: Direct Stage Heating 7

VI. Supplementary Note 6: COMSOL Simulations 8

VII. Supplementary Note 7: Hydrogenation 10
- A. SOI Raman Spectroscopy 10
- B. Float-Zone H Plasma Spectra 10
- C. Localized hydrogen incorporation 11

VIII. Supplementary Note 8: Electrical Analysis under Blanket Hydrogenation 12
- A. Hydrogen Plasma 12
- B. Ion Implantation 13

IX. Supplementary Note 9: G center annealing stability 14

X. Supplementary Note 10: Electrical Contact Annealing 15

XI. Supplementary Note 11: SRIM simulations 17

Supplementary References 18

* Equal Contribution

† Corresponding Author: ehu@seas.harvard.edu

I. SUPPLEMENTARY NOTE 1: ESTIMATION OF EMITTER-FIELD INTERACTION

The relevant role in the observed emitter interaction is the local electric field, rather than applied voltage. However, as we discuss in the text, intrinsic dopants in the ”I” layer of the junction inhibit typical field accumulation and prohibit complete junction depletion (as described in Anderson et al. Science 2019 [1], and Candido et al. PRX Quantum 2021 [2]), thereby rendering difficult the accurate determination of the local electric field experienced by the emitter. This is the reason for our spatially-dependent threshold voltage for Stark tuning, as was similarly observed in Anderson et al. Science 2019 [1].

We nonetheless can provide an estimate using the COMSOL junction simulations (Supplementary Note 6) and the Lorentz local field approximation (useful in instances where dopants do not interfere with the measurement, as in Lukin et al. NPJ Quantum Information 2020). Comparing our experimentally measured Stark tuning rate per applied voltage (1.24 GHz/V) to the field accumulation predicted by the COMSOL simulation (0.0098 (MV/m)/V) (Supplementary Figure 1) yields a tuning rate of 126.5 GHz/(MV/m). Now using the Lorentz local field approximation (see Lukin et al. NPJ Quantum Information 2020 [3])–where in silicon the local field is 4.56× greater than the applied field–the predicted Stark tuning rate is thus 27.75 GHz/(MV/m). We note that this predicted polarizability is on the same order of the 4.5-35 GHz/(MV/m) observed with the divacancy in silicon carbide observed by Anderson et al. Science 2019 [1].

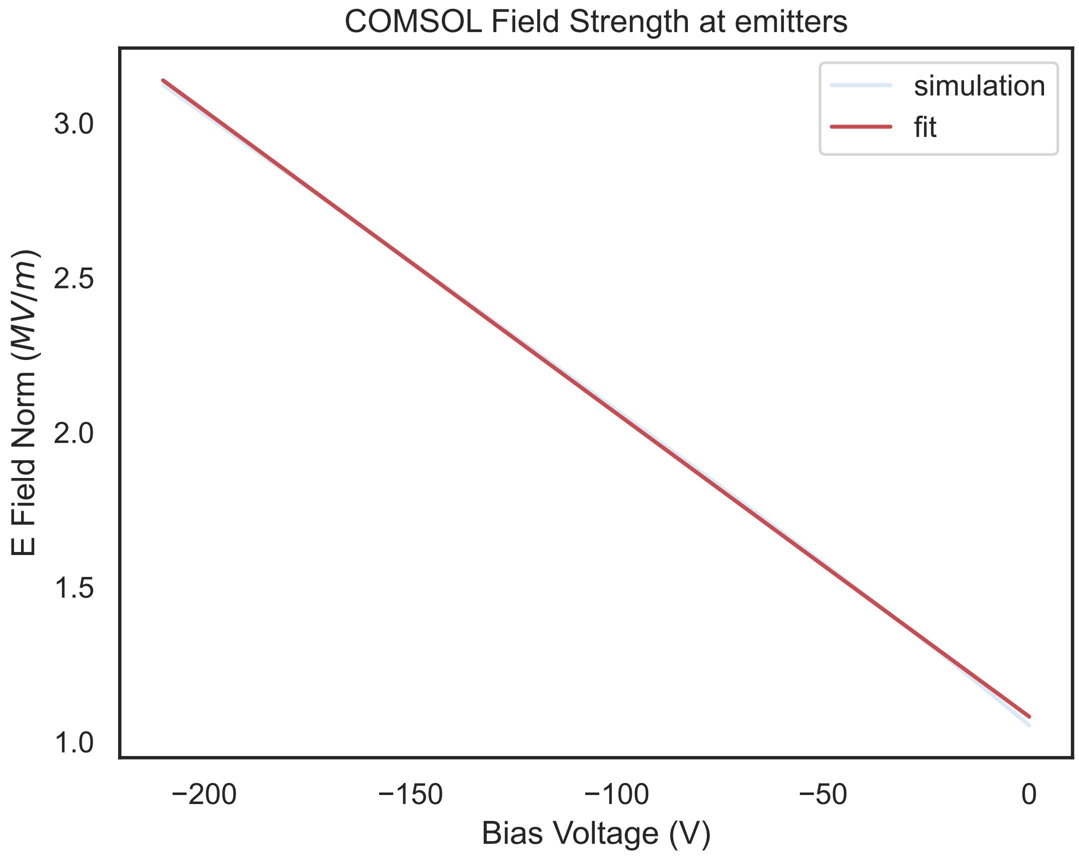

Supplementary Figure 1. **COMSOL Field Strength at Emitter (a.)** Voltage-evolution of the COMSOL-simulated electric field strength at the spatial location of the emitter where Stark tuning is experimentally observed

We opted not to include this estimate in the main text due to it’s failure to fully capture the background dopant-induced spatial distribution we (and others [1]) experimentally observe. For this reason, we continue to report our findings in terms of applied voltage, so that any other experimentalists performing similar measurements can use this as an accurate reference.

II. SUPPLEMENTARY NOTE 2: FITTING INFORMATION

The wavelength red-shift tuning rate given in the main text is found by fitting a Gaussian function to the emitter PL at every bias point, then extracting and plotting the center wavelength of the fitted curve. The entire dataset is given in Supplementary Figure 2. The raw data is shown in gray, where the Gaussian fit is the overlapped solid, colored, line–with color indicating reverse-bias voltage consistent with the main text.

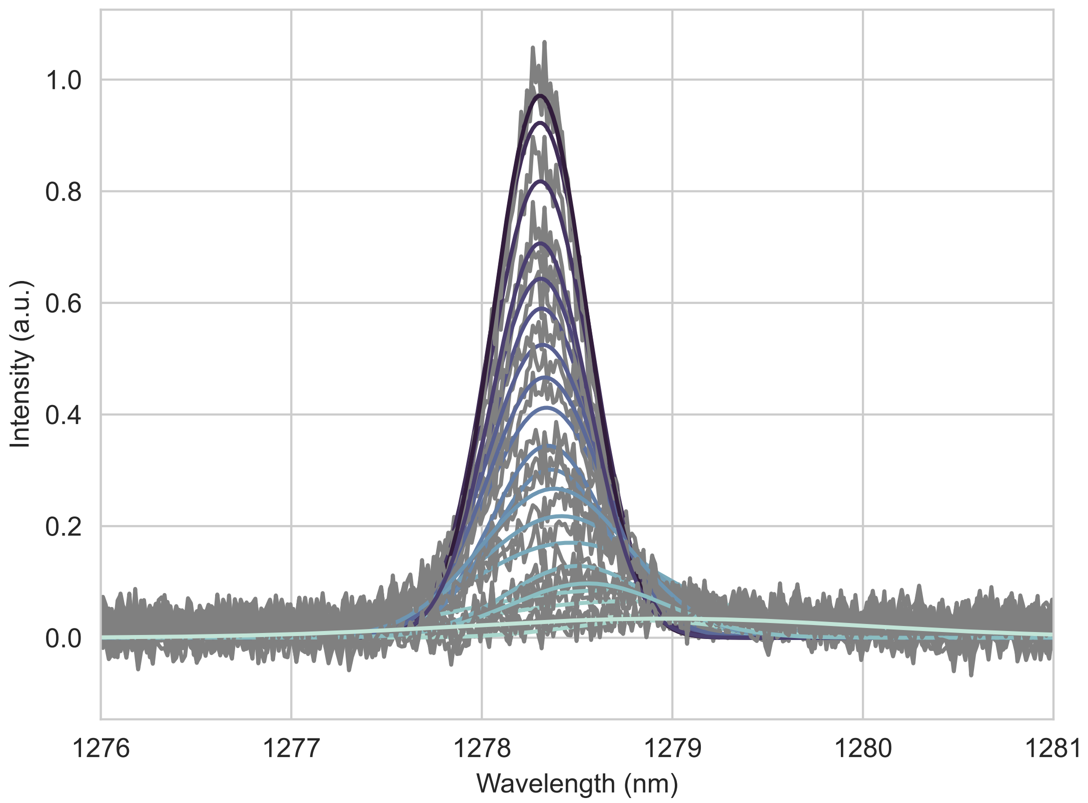

Supplementary Figure 2. **Stark-Effect Fitting (a.)** A Gaussian curve is fit to the measured emitter PL at every bias point. The center wavelength of the fitted function is then extracted and plotted to obtain a linear estimation of the Stark rate reported in the main text (Main Fig. 5b).

III. SUPPLEMENTARY NOTE 3: REVERSE BIAS EXTENDED DATA

A. Depletion Region

Here we present photoluminescence ensemble linewidth data as a function of applied voltage associated with that reported in the Main Fig. 3a, where redshift data are given in Main Fig. 5b. At approximately the same voltage where the ensemble begins to exprience a wavelength redshift, the ensemble experiences broadening in response to applied voltage (Supplementary Figure 3).

Although deviations from the trend are small in both Stark tuning (Main Fig. 5B), and linewidth (Supplementary Figure 3), one potential explanation could be the distribution of dipole orientations of individual emitters which comprise the ensemble. In [4], it was found that an investigated G center ensemble possessed a distribution of dipole orientations with occurrence clustering around 20° and 100° detuned from the $\langle 1\,1\,0 \rangle$ crystallographic axis. Therefore for a local electric field in the junction at the ensemble, the magnitude of the electric field coupling will vary depending upon the dipole alignment of the individual emitters which make up the ensemble, resulting in varied emitter detunings and overall broadening.

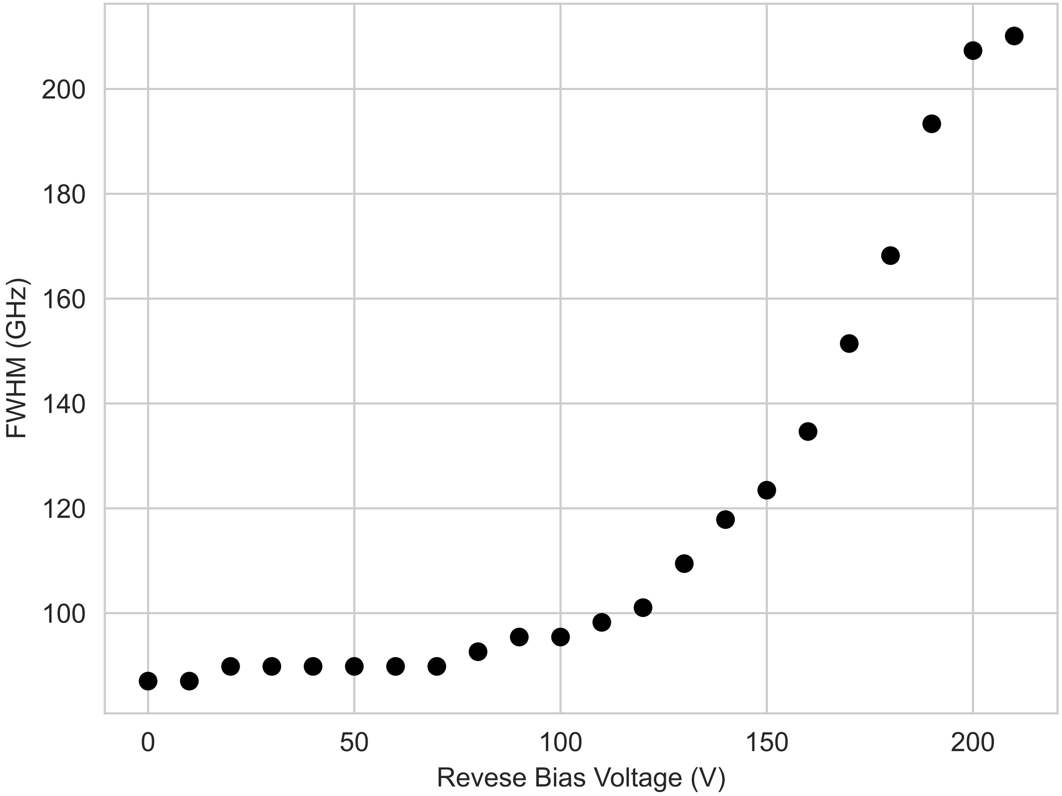

Supplementary Figure 3. **Reverse-bias within depletion region (a.)** Evolution of G center ZPL ensemble linewidth broadening from 0 to -210 V

B. Outside Depletion Region

Similarly, the photoluminescence redshift and ensemble linewidth of the partial extinction region shown in red in Main Fig. 5a is plotted in Supplementary Figure 4 as a function of applied voltage. The fluorescence intensity of the ensemble reduces while experiencing no meaningful wavelength tuning or broadening between zero and maximum reverse bias. This can be explained by the absence of a local electric field interacting with the ensemble to yield a Stark shift, as the depletion region has not yet reached the investigated spatial region far from the n-contact. However, emitter ionization is still facilitated under fermi level engineering and thus charge state modification of the emitters, resulting in the observed reduction in ensemble brightness.

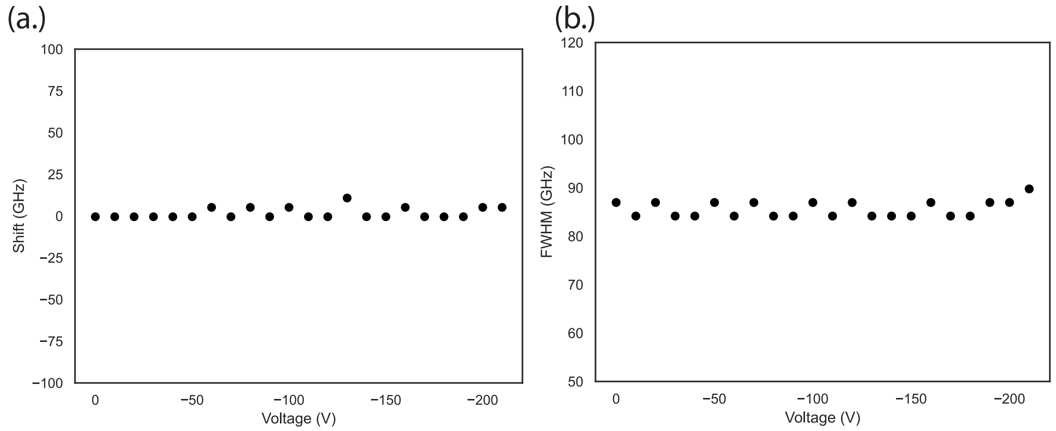

Supplementary Figure 4. **Reverse-Bias outside of depletion region** Evolution of G center ZPL **(a.)** center wavelength redshift, and **(b.)** linewidth broadening from 0 to -210 V

IV. SUPPLEMENTARY NOTE 4: FORWARD BIAS

In contrast to the emitter-field coupling illustrated under reverse bias (Main Fig. 3), in Supplementary Figure 5 we show that the optical response under forward bias–where orders magnitude greater current is passed– is dominated by local heating within the junction. The optical response of the same diode-integrated G center ensemble and silicon free-exciton is analyzed under application of a 0 to 60 V forward bias (limited to avoid device degradation at high current passage). Notably, G center ZPL and exciton experience *commensurate* modulation of the fluorescence intensity at increased forward bias–as to be expected at elevated local temperature. This finding is distinct from that of the reverse-bias results of the main text, where the silicon free-exciton is unperturbed while the ZPL experiences 100% modulation.

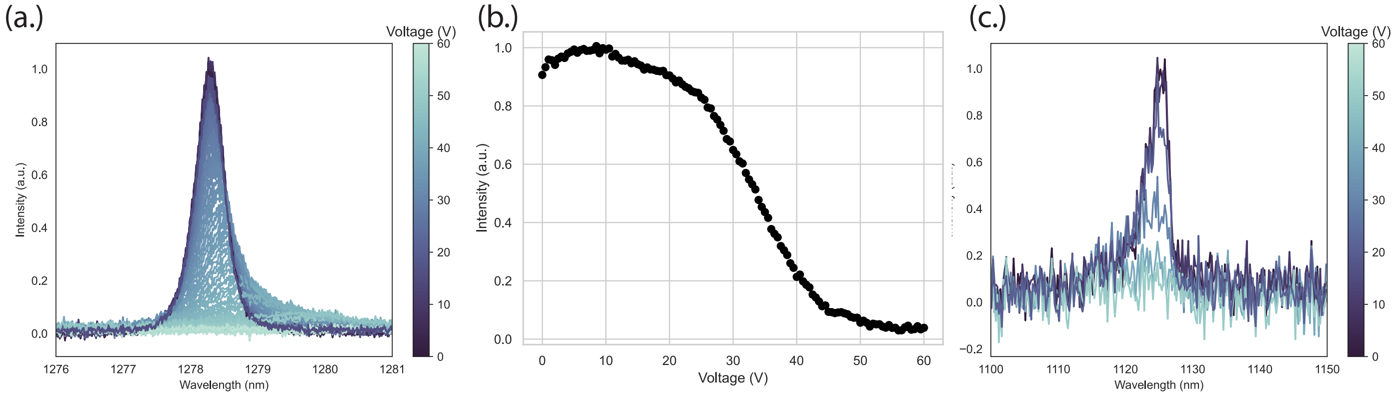

Supplementary Figure 5. **Forward-Bias response of G center ZPL and Silicon Free-Exciton** Evolution of **(a.)** G center ZPL with **(b.)** modulation per voltage. **(c.)** Silicon Free-Exciton under application of a forward-biased electric field from 0 to 60 V. As expected, both the ensemble and exciton experience commensurate suppression at high current passage, unlike in the reverse-biased regime.

Similarly, in Supplementary Figure 6, the G center redshift and linewidth evolution undergo markedly different qualitative responses when compared to the reverse bias case. Here ten-times greater linewidth broadening and wavelength-tuning is experienced at 4-times lesser absolute voltage. Under large forward bias power, the emitter-field coupling competes with local heating in the junction, resulting in ZPL broadening and shifting consistent with that observed under direct heating. These results capture the optimal electrical performance of the junction, and further illustrate the unique behavior observed above under reverse-bias in the main text.

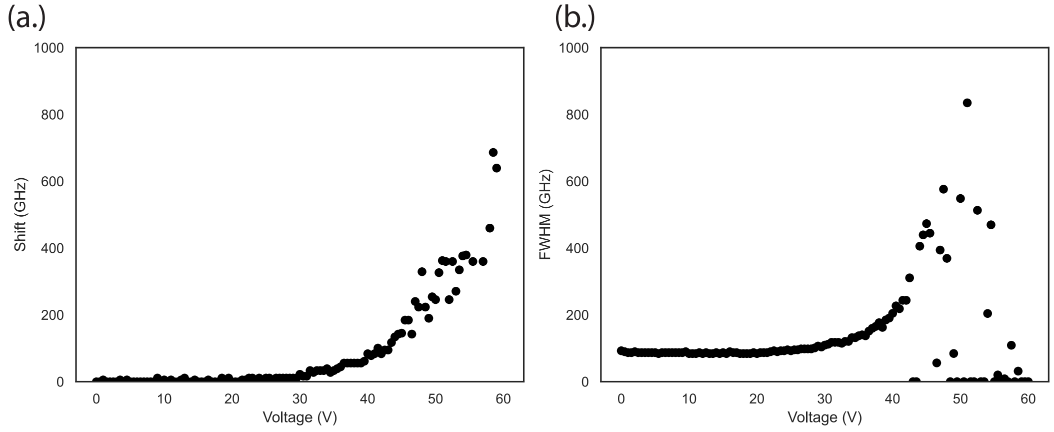

Supplementary Figure 6. **Wavelength Analysis of G center ZPL under Forward-Bias (a.)** center wavelength red-shift, and **(b.)** linewidth broadening, revealing order magnitude greater broadening and redshift occurring at lower absolute voltage than in the reverse-bias regime.

V. SUPPLEMENTARY NOTE 5: DIRECT STAGE HEATING

For reference, heating power is directly applied to the sample stage cold finger in the cryostat on a bulk reference sample (no electrical driving of diode-integrated emitters), and the G center ensemble photolumninescence is measured as a function of cryostat temperature (Supplementary Figure 7). The ZPL evolution follows notably similar reduction, broadening, and redshift as is reported above via forward biasing an electrical junction. Comparing the ZPL intensity and linewidth under direct sample stage heating to that of the forward biasing of electrical junctions, it appears that the ensemble is elevated to approximately 60 K. At 50 V forward bias, approximately 9 mA is passed through the junction–corresponding to 450 mW power. Therefore, we can estimate that forward-bias driving the junction with roughly 27 dBm locally heats the ensemble by 55 K.

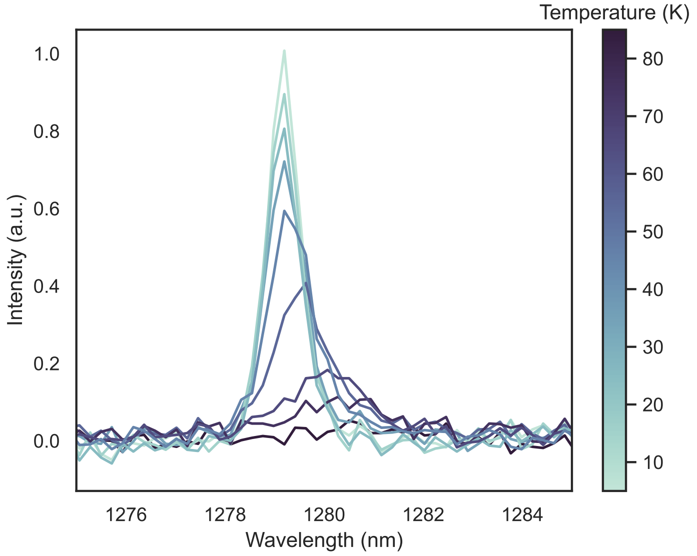

Supplementary Figure 7. **G center ZPL evolution under direct sample stage warming (a.)** G center ZPL evolution from roughly 4 K to 80 K. As a function of temperature, the ensemble optical efficiency, linewidth, and center wavelength undergo a simultaneous reduction, broadening, and redshift, respectively. At roughly 80 K, one second integration on the spectrometer is insufficient to resolve the emitter ZPL.

VI. SUPPLEMENTARY NOTE 6: COMSOL SIMULATIONS

A COMSOL model of the device presented in the main text is built and simulated (Supplementary Figure 8) using the semiconductor module to study the spatial dependence on the valence and conduction band bending, space charge, electric field strength in the diode in the region where the G center ensemble is located. The investigated device has a device size d–defined from center to center of the metal contacts–of 603 μm. Therefore the gap between doped apertures that the junction and ensemble occupy is 103 μm.

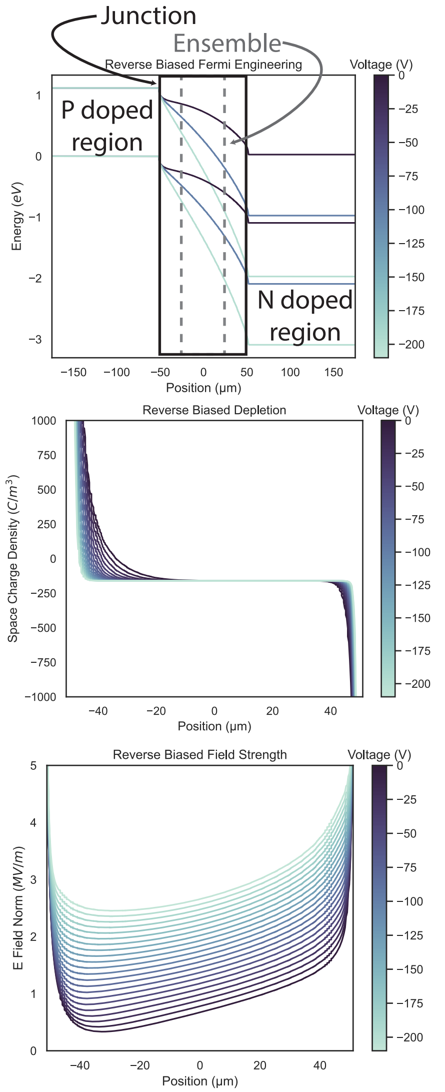

Supplementary Figure 8. **COMSOL simulation of Reported Device** COMSOL simulation analysis of junction behavior as a function of reverse bias voltage at the spatial region occupied by the G center ensemble. Data taken as a 1D slice of the device at a depth of 110nm (halfway through the 220nm device layer). **(a.)** Valence and conduction band edge bending, **(b.)** space charge density, and **(c.)** electric field norm.

The simulations present qualitative trends which agree with our experiment, whereby the embedded ensemble interacts with the device through numerous effects. Namely:

- The valence and conduction bands bend across the junction. We observe this via the brightness gradient induced by charge state modification of the emitters.
- The space charge region is asymmetric, due to the built-in natural depletion layer near the N contact by nature of the lightly p-doped background host material. Additionally, as increasing reverse bias is applied, the depletion region widens. We observe this via the spatial distribution and evolution of the confocally-excited photocurrent.

- The electric field norm is higher closer to the N contact. We observe this via the spatial distribution of emitter Stark tuning, which begins near the N contact.

However, exact quantitative agreement with simulation–where local electric field and junction depletion does not reach the ensemble until a sufficient threshold voltage–is not directly evident in the simulation, as the model less clearly captures the spatial distribution of residual background dopants. A more accurate simulation of these effects is calculated from first principles in [2]–to validate similar experimental [1] findings as ours–in which the relevant physics are more fully captured than with a simple COMSOL model.

VII. SUPPLEMENTARY NOTE 7: HYDROGENATION

Here we investigate optimal means of G center formation and device integration. The optical and electrical properties of the devices and the formed emitters are studied under varied methods of hydrogen inclusion. Specifically, we vary the hydrogen ion implantation densities, and then compare with samples exposed to a hydrogen plasma of incremental duration ranging from zero to two minutes. The plasma is generated in a reactive ion etching chamber solely comprised of H_2 gas under 35 mTorr pressure, a flow-rate of 30 sccms, and using 1000 W coil and 200 W platen powers.

A. SOI Raman Spectroscopy

To understand the extent of hydrogen incorporation during implantation versus plasma treatment, the Raman signatures of samples that underwent each treatment are shown in Supplementary Figure 9. All samples were first carbon ion implanted and rapid thermal annealed at 1000 C for 20 s. One sample additionally possessed the diode devices discussed throughout this work. The Raman spectra of all investigated samples have the same basic features with the exception of either the presence or absence of a peak at $2100 cm^{-1}$. This peak has previously been identified as characteristic of a silicon-hydrogen (Si-H) stretch-bond present on sample surfaces [5]. This Si-H stretch bond line is evident in all samples which were exposed to the hydrogen plasma, even those which received the shortest investigated exposure time of 30 seconds. However, this peak was absent from the samples which received hydrogen implantation and those which were untreated, suggesting the plasma treatment uniquely modifies the wafer surface.

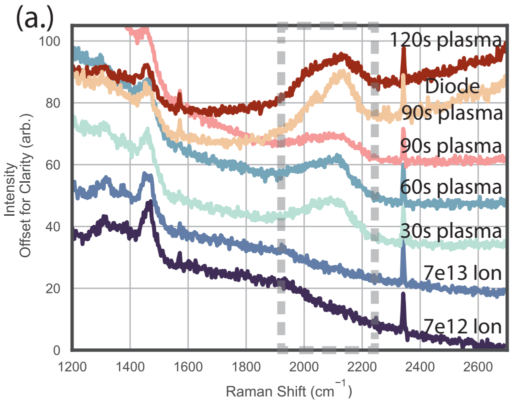

Supplementary Figure 9. **SOI Raman (a.)** Raman spectra comparing hydrogen ion implantation to hydrogen plasma exposure across SOI samples. The $2100 cm^{-1}$ Si-H stretch bond (dotted-gray boxed region) is only present for samples which received hydrogen plasma exposure.

B. Float-Zone H Plasma Spectra

The effect of hydrogen implantation versus plasma treatment is also investigated on electron-irradiated float-zone (FZ) silicon samples. Similarly, the $2100 cm^{-1}$ peak occurs only in samples that underwent hydrogen plasma exposure (Supplementary Figure 10).

Additionally, In Supplementary Figure 11 we report the correlated observation of G center PL in FZ samples which received the above hydrogen plasma treatment. These findings indicate that surface-related hydrogenation is sufficient to enable optical observation of G centers. Previous reports have considered bulk proton irradiation, however here we show that even a 30 second exposure of a surface treatment renders observation of G centers.

We thus establish both the correlation of the Si-H Raman band with hydrogen plasma exposure, and the ability to form G centers with both bulk and surface hydrogenation. Taken together, these results suggest that hydrogenation may play a central role in forming or stabilizing G center emission. However, further investigation is required to understand the contributory atomic-scale dynamics and mechanisms. For instance, though the plasma treatment is shown to be a surface-related effect by the Raman signature, it is not yet clear whether physical or chemical mechanisms render the formation of G centers under hydrogen plasma exposure–as plasma-induced knock-out could still mediate the incorporation of interstitial carbon for shallow G center formation on the FZ surface.

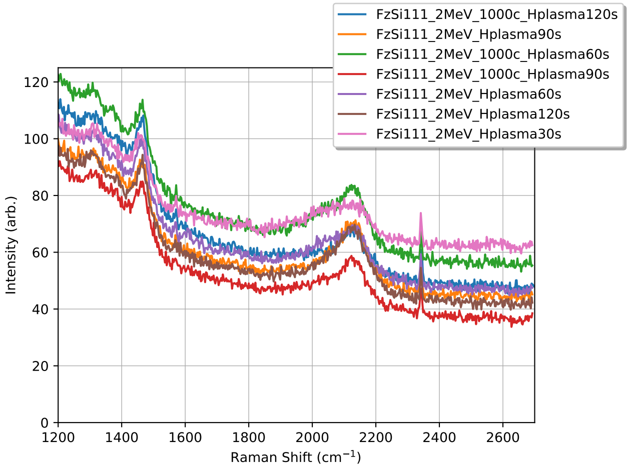

Supplementary Figure 10. **Float Zone Si Raman (a.)** Evidence of Si-H surface raman bond in float zone silicon analyzed under varied plasma exposure

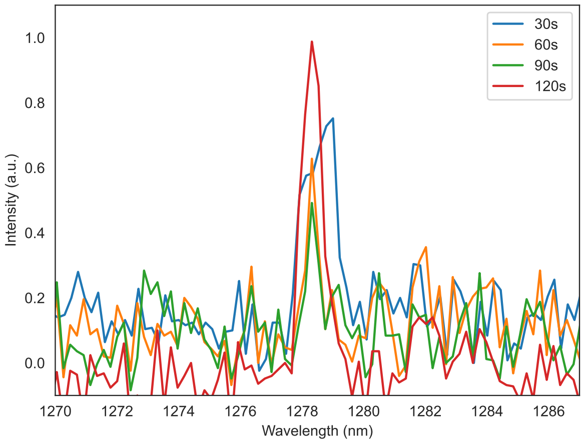

Supplementary Figure 11. **Float Zone Si PL (a.)** Associated G center PL of FZ samples exhibiting the Si-H raman bond.

C. Localized hydrogen incorporation

Though the presence of hydrogen was found to be critical to generate G centers, the interaction of hydrogen with the fabricated devices must also be considered. Significant electrical degradation is observed in diodes which received either blanket hydrogen ion implantation or blanket plasma exposure (see Supplementary Information Section 8). Junction resistance increased as a function of hydrogen plasma exposure, as shown in the IV-curves of Supplementary Figure 12. Further, the blanket-implanted devices no longer passed appreciable current at cryogenic temperatures Supplementary Figure 13. The utilized p-dopant, boron, is known to be passivated by hydrogen, and similarly, the conductivity of the gold contacts is expected to degrade under hydrogenation. To avoid these issues, a mask defined by optical lithography is ultimately employed for the utilized paper devices to achieve the formation of the desired emitters within the fabricated devices while preserving the electrical behavior of the diodes.

VIII. SUPPLEMENTARY NOTE 8: ELECTRICAL ANALYSIS UNDER BLANKET HYDROGENATION

In the main text, it was reported that masked hydrogenation was required to render simultaneous observation of G centers and the preservation of the electrical performance of the diodes. Here we report the data observed under various studies which motivated this decision.

A. Hydrogen Plasma

The electrical properties of diodes are investigated under incremental exposure to a hydrogen plasma. A continual reduction of current passage–and seemingly increase in contact resistance–is observed for each subsequent exposure in every investigated device. Supplementary Figure 12a shows room temperature IV-curves of four devices under 30 second increments of the hydrogen plasma exposure discussed in the main text, ranging from 0-60 seconds. In Supplementary Figure 12b, we repeat this process on four new devices, this time allowing a full minute of exposure, such that these devices received first a 30 s and then a 60 s exposure. The peak current passage is uniformly reduced for all investigated conditions on all evaluated devices, consistent with an increase in contact resistance, and potential passivisation of boron dopants in the junction.

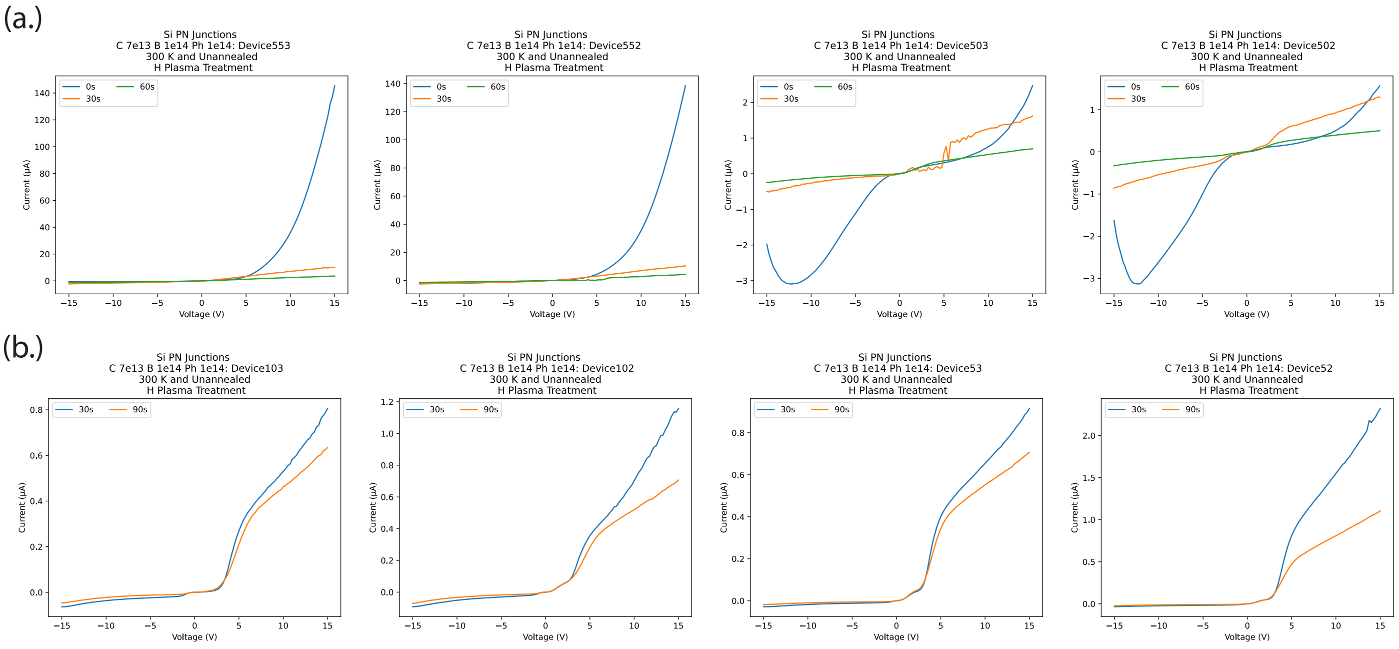

Supplementary Figure 12. **IV-analysis under H plasma** Consistent degradation of diode characteristics under increased hydrogen plasma exposure, evidence across every investigated device. Evolution of eight different devices are given, with **(a.)** successive 30 second treatments, and **(b.)** a 30 second treatment followed by a 60 second treatment.

B. Ion Implantation

Diodes which were blanket implanted with hydrogen ions showed a similar degradation of their electrical properties following implantation as is evident under incremental plasma treatment. However, upon a 4 K cooldown cycle, the devices suffered permanent damage where appreciable current is no longer passed at all (Supplementary Figure 13). The pre-cooldown operation of the device is not recovered upon warm-up, and thus it appears that the process of cooling the bulk hydrogenated diodes imparts permanent damage–perhaps due to contact delamination under thermal expansion and contraction upon hydrogenating the Ti-Au.

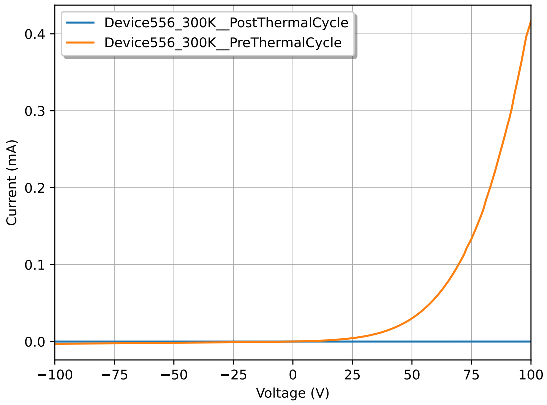

Supplementary Figure 13. **Ion implanted IV-analysis under cooling cycle (a.)** Diodes which received a blanket hydrogen ion implantation suffered permanent damage upon a thermal cycle in the cryostat. Furthermore, current passage is heavily reduced (0.4 mA at 100 V) compared to the device of the main text which received a masked implant.

IX. SUPPLEMENTARY NOTE 9: G CENTER ANNEALING STABILITY

We analyze the stability of optical emission of G center ensemble under varied annealing conditions to best evaluate emitter integration in the fabricated electrical devices. Here the investigated samples are all identically co-processed up until the thermal anneal, consistent with the emitter synthesis outlined in the main text. Then, each sample is placed on a hot plate at 400°C for incremental times. Notably, we observe complete loss of optical emission of the ensemble in as short at 30 seconds of heating (Supplementary Figure 14).

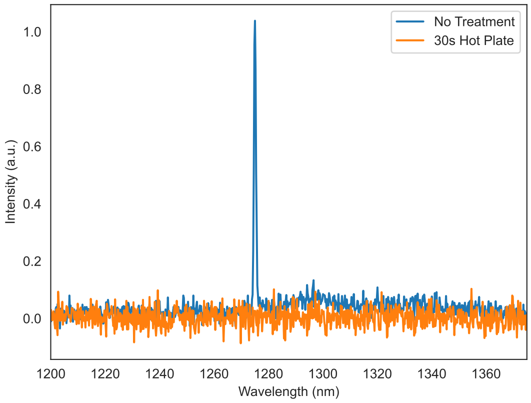

Supplementary Figure 14. **Time-dependent analysis of rapid thermal anneal (a.)** All investigated times of exposure to a 400°C hot plate resulted in quenched G center emission, even in as little as 30 seconds. A 4×4 mm wafer sample was directly placed on a thermally-equilibriated hot plate for exactly 30 seconds, then removed and allowed to naturally cool.

Additionally, we repeat the experiment on samples held at lower temperatures and find that G center emission is unaffected. We observe preserved optical emission in samples held on a hot plate at 115°C for 3min, and at 180°C for 3min. These two temperatures and times are investigated due to their association with with resist mask baking for optical and electron-beam lithography, respectively.

In summary we find G center optical emission to be stable on samples annealed up to 180°C, but unstable at 400°C under as little as a 30 s anneal. These findings are consistent with those reported in [6], where they find G center optical emission to be unstable above 200°C. For this reason, incorporation of G centers using hydrogen is performed as the final step in the fabrication process. Similarly, future work using samples which received a contact-anneal will incorporate hydrogen following the contact anneal, as gold contact annealing temperatures (investigated in Supplementary Note 10) are around 400°C.

X. SUPPLEMENTARY NOTE 10: ELECTRICAL CONTACT ANNEALING

The electrical performance of our fabricated diodes is investigated under thermal annealing at varied temperatures, with the findinds summarized in Supplementary Figure 15, taken from the data of Supplementary Figure 16. The thermal anneal is expected to improve the ohmic nature of the Ti-Au electrical contacts, and thus four different conditions are evaluated. Each anneal was performed in a tube furnace under vacuum, with the target temperature held for 2 minutes. The pressures of each anneal, from 350 °C to 400°C, were 7.1e-6, 8.2e-6, 9.5e-6, 6.6e-6 Torr, respectively. It took approximately 9 minutes for the furnace to ramp from 20°C to target temperature, and the sample cooled down naturally after the 2 minute anneal. We characterize the degree of improvement achieved via anneal by the maximum current passed through the junction for the given IV-sweep that was selected (-15 to 15 V), as enhanced electrical contact between the dopants and the electrode will result in lower contact resistance.

We classify the response of our devices between *short* and *long* PN junctions, where *short* refers to devices with a junction width between doped pads less than 50 μm. The long junctions systematically out-perform the short junctions, including in the degree of enhancement found under thermal anneal. We suspect the reason for this is that for a small gap between the doped regions, mask alignment errors from the successive aligned optical lithography writes likely result in a partial overlap of the P and N dopant aperatures, resulting in poor diode performance.

Ultimately, the device used in this work (a 103 μm gap *long* junction) received no contact anneal, as it's performance was sufficient to observe the desired emitter-field interaction. However, future works would benefit from performing a 2 minute thermal anneal at 400°C prior to hydrogen incorporation.

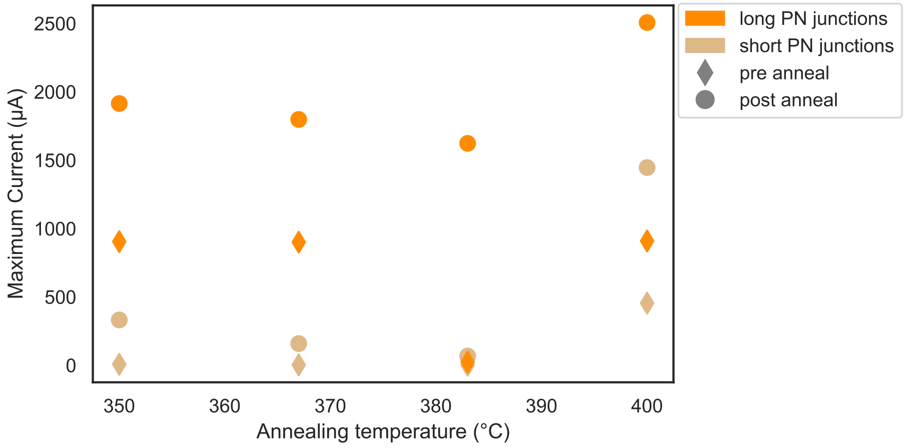

Supplementary Figure 15. **Summary of contact anneal enhancement (a.)** Diodes which received a contact anneal exhibited uniform improvement in their IV-characteristics at all investigated temperatures from 350-400°C. All investigated devices show improvement from anneal, and the degree of enhancement is approximately 2× for most of the investigated devices.

Additionally, we note that the T center in silicon has been reported to be observed following a comparable annealing condition as was investigated here for the electrical contacts. As such, it would be interesting to investigate the diodes under the reported [7] T center process of:

1. hydrogen ion implantation
2. 1 hour boil in deionized water
3. ∼ 400°C rapid thermal anneal for simultaneous T center synthesis and contact annealing.

to find if both the electrical properties of the diodes improve and if T centers are observed. However, in our background investigations with bulk SOI, T centers are not observed following the reported recipe.

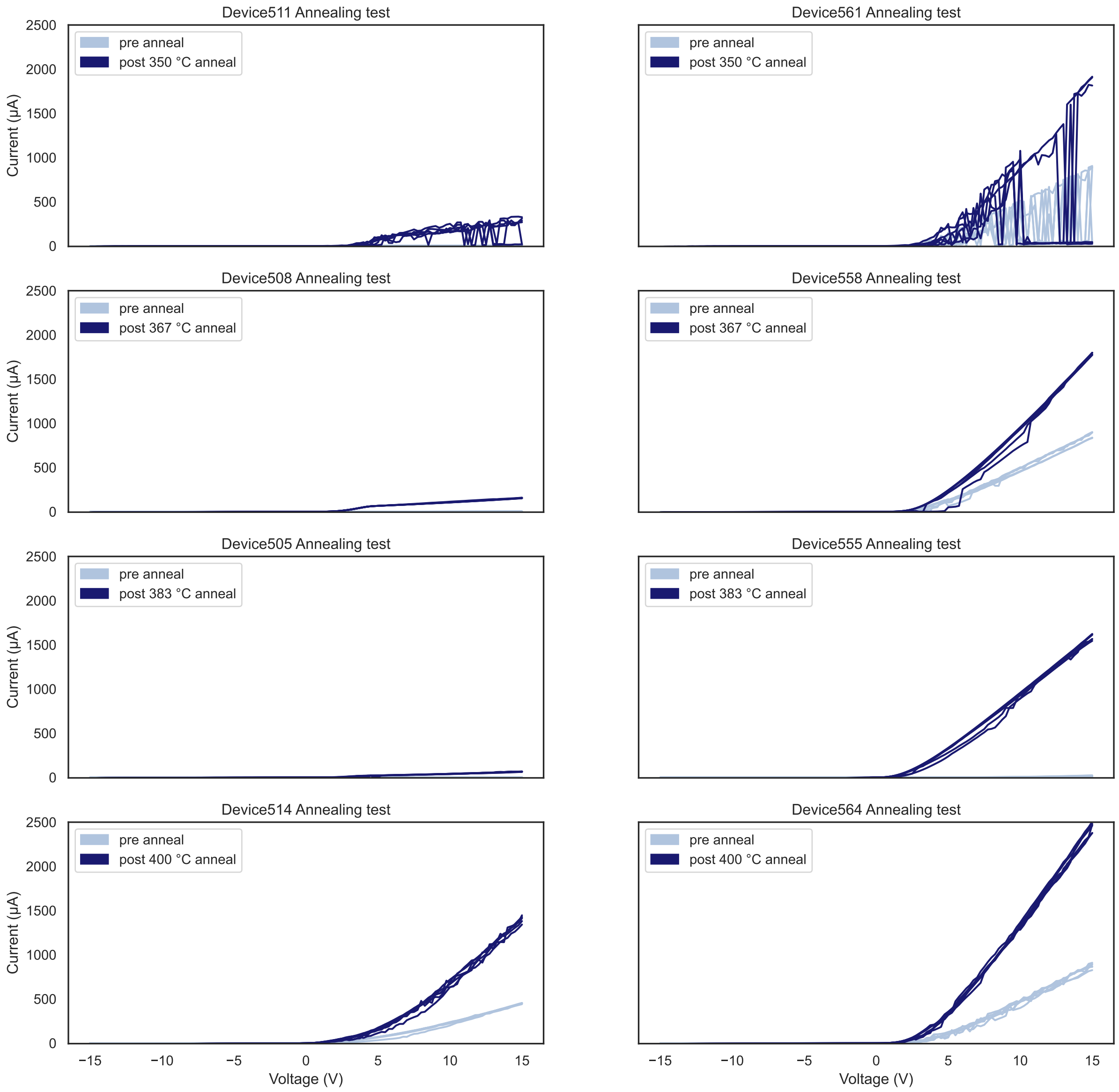

Supplementary Figure 16. **Detail of contact anneal (a.)** IV curves are measured before and after a contact anneal at four different temperatures for eight different devices. The left column are *short* junctions, where the right column are *long* junctions. The data of these curves are summarized in Supplementary Figure 15

XI. SUPPLEMENTARY NOTE 11: SRIM SIMULATIONS

Stopping range of ions in matter (SRIM) simulations are performed for the four ion species used in this work to find the optimal energies and doses to incorporate boron, phosphorus, carbon, and hydrogen in the same spatial plane and at desired densities.

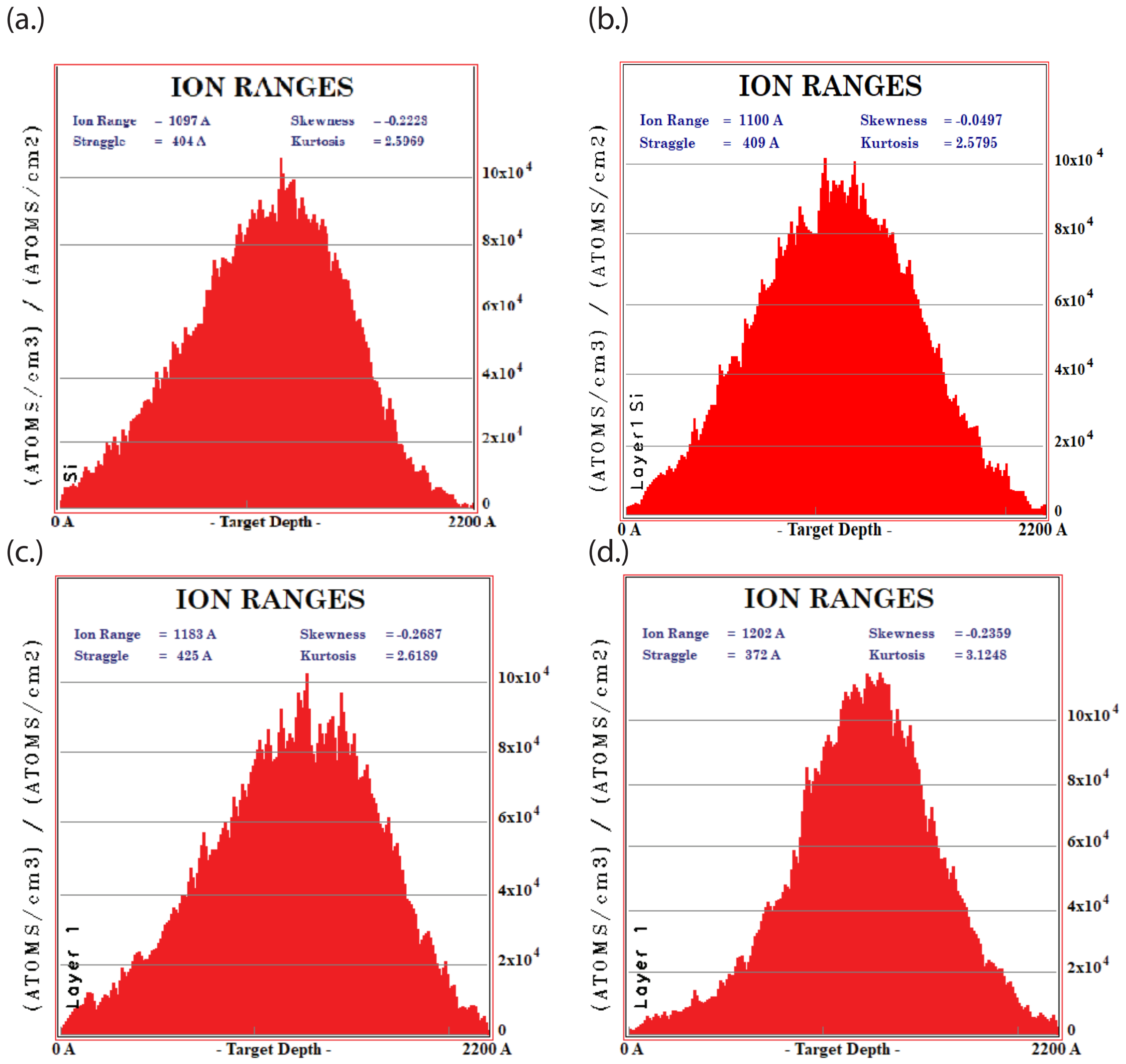

Supplementary Figure 17. **SRIM simulations of utilized ions** SRIM calculations to enable a stopping distance at 110 nm using **(a.)** boron, **(b.)** phosphorus, **(c.)** carbon, and **(d.)** hydrogen.

[1] C. P. Anderson, A. Bourassa, K. C. Miao, G. Wolfowicz, P. J. Mintun, A. L. Crook, H. Abe, J. Ul Hassan, N. T. Son, T. Ohshima, *et al.*, Electrical and optical control of single spins integrated in scalable semiconductor devices, Science **366**, 1225 (2019).

[2] D. R. Candido and M. E. Flatté, Suppression of the optical linewidth and spin decoherence of a quantum spin center in a p-n diode, PRX quantum **2**, 040310 (2021).

[3] D. M. Lukin, A. D. White, R. Trivedi, M. A. Guidry, N. Morioka, C. Babin, Ö. O. Soykal, J. Ul-Hassan, N. T. Son, T. Ohshima, *et al.*, Spectrally reconfigurable quantum emitters enabled by optimized fast modulation, npj Quantum Information **6**, 80 (2020).

[4] W. Redjem, Y. Zhiyenbayev, W. Qarony, V. Ivanov, C. Papapanos, W. Liu, K. Jhuria, Z. Al Balushi, S. Dhuey, A. Schwartzberg, *et al.*, All-silicon quantum light source by embedding an atomic emissive center in a nanophotonic cavity, Nature Communications **14**, 3321 (2023).

[5] M. Brodsky, M. Cardona, and J. Cuomo, Infrared and raman spectra of the silicon-hydrogen bonds in amorphous silicon prepared by glow discharge and sputtering, Physical Review B **16**, 3556 (1977).

[6] L. Canham, K. Barraclough, and D. Robbins, 1.3-μm light-emitting diode from silicon electron irradiated at its damage threshold, Applied physics letters **51**, 1509 (1987).

[7] E. MacQuarrie, D. Higginbottom, K. Morse, and V. Karasyuk, Generating t centres in photonic silicon-on-insulator material by ion implantation, New Journal of Physics **23**, 103008 (2021).
